# Indoor Air Quality Monitoring Systems Based on Internet of Things: A Systematic Review

**DOI:** 10.3390/ijerph17144942

**Published:** 2020-07-09

**Authors:** Jagriti Saini, Maitreyee Dutta, Gonçalo Marques

**Affiliations:** 1 National Institute of Technical Teacher’s Training and Research, Chandigarh 160019, India; jagritis1327@gmail.com (J.S.); d_maitreyee@yahoo.co.in (M.D.); 2 Instituto de Telecomunicações, Universidade da Beira Interior, 6200-001 Covilhã, Portugal

**Keywords:** indoor air quality, Internet of Things, monitoring systems, public health

## Abstract

Indoor air quality has been a matter of concern for the international scientific community. Public health experts, environmental governances, and industry experts are working to improve the overall health, comfort, and well-being of building occupants. Repeated exposure to pollutants in indoor environments is reported as one of the potential causes of several chronic health problems such as lung cancer, cardiovascular disease, and respiratory infections. Moreover, smart cities projects are promoting the use of real-time monitoring systems to detect unfavorable scenarios for enhanced living environments. The main objective of this work is to present a systematic review of the current state of the art on indoor air quality monitoring systems based on the Internet of Things. The document highlights design aspects for monitoring systems, including sensor types, microcontrollers, architecture, and connectivity along with implementation issues of the studies published in the previous five years (2015–2020). The main contribution of this paper is to present the synthesis of existing research, knowledge gaps, associated challenges, and future recommendations. The results show that 70%, 65%, and 27.5% of studies focused on monitoring thermal comfort parameters, CO_2_, and PM levels, respectively. Additionally, there are 37.5% and 35% of systems based on Arduino and Raspberry Pi controllers. Only 22.5% of studies followed the calibration approach before system implementation, and 72.5% of systems claim energy efficiency.

## 1. Introduction

Indoor air pollution (IAP) is a leading environmental risk closely related to the health, comfort, and well-being of building occupants [1]. As people spend 90% of their time indoors, repeated exposure to indoor air pollutants affects people’s working performance and productivity levels [2]. It has been reported as a potential cause behind the loss of USD 20 to 200 billion per year due to a 0.5 to 5% decrease in workplace productivity [3]. The impact of IAP can be up to 100 times higher as compared with outdoor pollutant levels [4]. This is because closed spaces promote the build-up of potential pollutants with considerably higher efficiency than open spaces. One half of the global population and 95% of people in low- and middle-income countries rely on solid fuels such as biomass and coal for their routine cooking and heating needs [5]. In India, 0.2 billion people make use of fuel for cooking, out of which 49% rely on firewood; 28.6% prefer liquid petroleum gas, 8.9% use cow dung cake; 2.9% use kerosene, 0.4% biogas, 0.1% electricity, and 0.5% use other alternative means [6]. The incomplete combustion of biomass fuels in traditional stoves, especially in poorly ventilated homes, leads to higher levels of carbon monoxides (CO), particulate matter (PM), formaldehyde, nitrogen oxides (NOx), polycyclic aromatic hydrocarbons, benzene, and other toxic organic compounds, which further leads to chronic health problems [5].

The impact of IAP is not limited to rural homes. The scientific community reveals that indoor air quality (IAQ) has been a dynamic and complex issue for modern housing arrangements in urban areas. The concentration of pollutants rises due to several internal sources, including building materials, heating, ventilation and air conditioning (HVAC) systems, use of chemical-rich products, and other human activities [7]. Air pollution levels are extremely influenced by frequent activities of hospital staff in patient care wards, use of chemical compounds at pharmacies and laboratories, and due to the use of harmful disinfectants in living spaces. The risks of IAP levels are high for children, elderly people, disabled patients, and office employees that stay indoors for an extended period. The ill-effects of IAP contribute to 2 million premature deaths annually, out of which 2% die from lung cancer, 54% from chronic obstructive pulmonary disease (COPD) and 44% die because of pneumonia [6]. It is also a potential cause behind rising threats of respiratory health problems [8]. These problems include low birth weight [9], stillbirth [10], lung cancer [11], and acute respiratory tract infection [12]. Despite the lifestyle habits, and heating and cooking system preferences in the developed and developing countries over the years, IAP has been a potential cause behind rising morbidity and mortality rates [13,14]. In order to control the harmful impacts of polluted indoor environments on building occupants, it is crucial to harness the potential of the latest technologies [2]. Researchers around the world have designed and developed IAQ monitoring systems to provide real-time updates regarding threatening IAP levels. However, the effective use of these systems to address relevant challenges in field environments is still a matter of concern [15].

**Figure 1:**
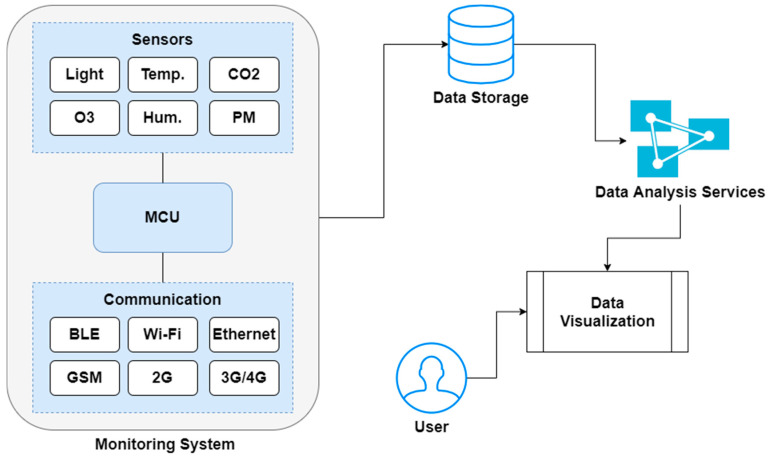
General architecture of Internet of Things (IoT)-based IAQ monitoring systems.

There are two potential technologies that present a solid platform for the development of IAQ monitoring systems: wireless sensor technologies (WSN) and Internet of Things (IoT) [15]. As the latest government policies are promoting the development of smart cities and smart villages with the influence of IoT-based architectures, it is relevant to analyze the potential of IoT for real-time IAQ monitoring applications. The combination of IoT with new-age information and communication technologies promises reliable solutions for enhanced environmental health and well-being [16]. These monitoring systems include two relevant components: hardware and software. These domains work together to provide instant updates regarding pollutant levels. On the one hand, the selection of the right sensors, microcontrollers (MCUs), and gateways is a crucial factor for upcoming researchers. On the other hand, communication technologies such as Wi-Fi, ZigBee, Bluetooth, and Ethernet are used for real-time updates regarding pollutants concentrations [17]. Moreover, as most of the existing systems are evaluated and installed in laboratory settings or controlled environments, reliable decision-making, assessment, and measurement of field IAQ parameters are still a challenging task. It is crucial to create a sustainable approach to address the problems associated with IAP while promoting citizen’s health with affordable solutions. The architectures, communication technologies, and hardware requirements must be analyzed in-depth to handle sensible data associated with routine activities of building occupants. Figure 1 describes the general architecture of IoT-based IAQ monitoring systems. The structure is mainly divided into four parts: monitoring system, data storage, data analytics services, and data visualization system. The monitoring system includes various IAQ sensors, MCUs, and communication systems. The data collected via a sensing unit is further stored into a data storage system that can be an online storage or physical storage. Furthermore, the data analytics services can be employed to analyze the impact of pollutants in the target premises. The visualization system further helps end users to get instant updates about IAQ levels.

This systematic review paper provides insights into the current state of the art of IoT-based IAQ monitoring systems that have been developed within the last five years (2015–2020). This study helps to analyze and synthesize essential details about existing systems, along with their hardware and software components for enhanced living environments. The main aim of this paper is to present a review of the widely preferred system architectures, sensor units, MCUs, connectivity options, and communication protocols. The synthesized information describes gaps in the existing body of knowledge while highlighting potential challenges and limitations of existing systems. This paper also provides recommendations and guidelines for future research directions to enhance public health and well-being. This study influences the wide adoption of the smart building concept while promising enhanced monitoring and assessment of IAQ levels.

## 2. Research Methodology

This systematic review was done in accordance with the PRISMA (Preferred Reporting Items for Systematic Review and Meta-Analysis) checklist. The process was broken down into several steps to address the challenges associated with real-time IAQ monitoring applications based on IoT. Relevant research questions were identified at the first step, and then the search strategy was described along with specific search strings and keywords. The inclusion and exclusion criteria were defined to assist with the selection of the most relevant publications from existing databases. Next, data extraction and synthesis were carried out on the basis of pre-defined research questions. Furthermore, the Results section describes a detailed analysis of the existing state of the art of IoT-based IAQ monitoring systems while highlighting potential challenges, limitations, and opportunities. The steps for conducting this systematic review are defined in detail in the following subsections.

### 2.1. Research Questions

The main objective of this systematic review is to provide an overview of the field of IAQ monitoring while highlighting potential gaps in the knowledge for upcoming researchers. Therefore, this paper can serve as a guide for researchers that are interested in developing real-time monitoring systems to address public health challenges associated with IAP. The following research questions (RQs) were identified, and a detailed analysis was provided to answer:

RQ1: What are the different types of sensors used for IAQ monitoring?
RQ2: What are the parameters supervised by existing researchers?
RQ3: What are the MCUs used to connect these sensors?
RQ4: What are the preferred interfaces for air quality sensing?
RQ5: What are the preferred communication technologies?
RQ6: What are the power requirements and energy efficiencies of the existing systems?
RQ7: What are the functionality details of the implemented IAQ monitoring systems?

With RQ1 and RQ2, we are able to identify different types of sensors used by existing researchers and potential parameters measured for IAQ monitoring. RQ3 and RQ4 provide insights into the preferred architectures for connecting these sensors while describing details about widely used MCUs and interfaces. Furthermore, RQ5 allows us to know about most preferred communication technologies to ensure real-time monitoring in indoor premises. Finally, RQ6 and RQ7 provide information about power requirements, energy efficiency, calibration requirements, and other crucial functionality details for field implementations.

### 2.2. Search Process

For conducting this systematic review, four literature databases were used to identify relevant publications about existing IAQ monitoring systems. These four databases are Web of Science, IEEE Explore, ScienceDirect, and PUBMED. The research for relevant publications on these databases was initiated on 8 May 2020. The search results were further exported to the Sysrev platform that provides direct insights into publication titles and abstract. The selected publications were further imported to the Zotero reference manager.

The initial search query for executing this systematic review was defined as the following combination of keywords:

*“indoor air quality” AND “monitoring systems” AND “sensors” AND “Internet of Things”*

**Figure 2:**
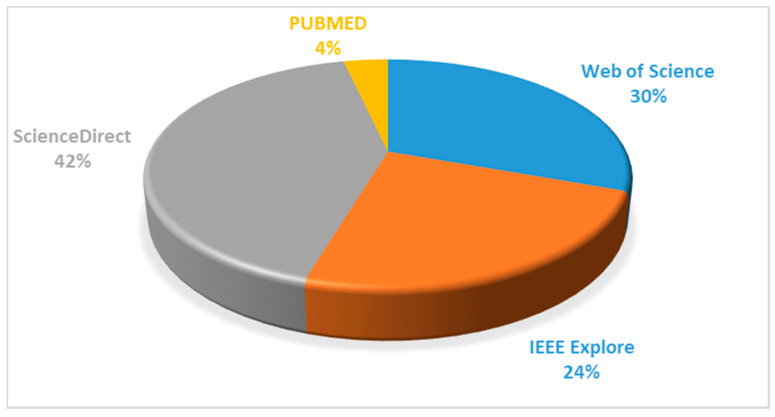
Distribution of studies obtained from four databases after the initial search query.

The search process resulted in 141 publications from all four databases, out of which 43 entries were identified from Web of Science, 59 were obtained from ScienceDirect, and 34 entries were listed from IEEE Explore, whereas only 5 publications were obtained from the PUBMED database. Figure 2 shows the percentage distribution of studies from different databases.

### 2.3. Inclusion and Exclusion Criteria

The eligibility criteria for the selection of relevant literature are composed of inclusion and exclusion criteria. As mentioned in Section 1, this systematic review is focused on IoT-based IAQ monitoring systems. Therefore, WSN-based systems were excluded from this study. Table 1 describes the inclusion and exclusion criteria for the selection of the most relevant literature for conducting this systematic review.

### 2.4. Study Selection

**Figure 3:**
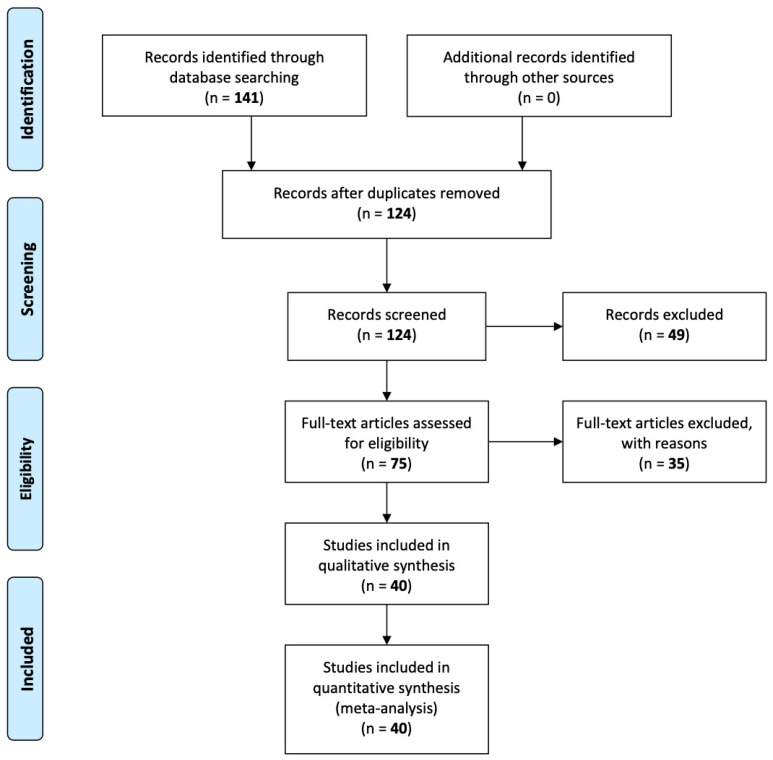
PRISMA flow diagram for systematic review.

The initial search query was applied to four databases that resulted in 141 publications. All these publications were first transferred to the Sysrev platform, and then duplicate papers were excluded based on PRISMA guidelines. Following EC1, 17 duplicate publications were removed, and the remaining 124 publications were used for the next level of screening. Following the above-mentioned inclusion and exclusion criteria, 49 documents were removed from the list, and the remaining 75 papers were imported for full-text screening. In total, 35 studies were considered irrelevant at this stage, out of which eight [18,19,20,21,22,23,24,25] were removed due to missing details about IoT (EC1). Two [26,27] were excluded following EC4 as they were review papers or theoretical analysis (EC3, EC4). Furthermore, 20 studies [28,29,30,31,32,33,34,35,36,37,38,39,40,41,42,43,44,45,46,47] were removed due to missing information about the design methodology and the type of sensors used for measuring IAQ parameters (IC4, EC3). In addition, five more studies [48,49,50,51,52] were excluded as they were focused on thermal comfort parameters only or had no relevant details about IAQ sensors (IC2, IC3, EC3). After applying inclusion and exclusion criteria, 40 studies were found eligible for conducting this systematic review. Clear insights into the selection process as per the PRISMA flow diagram are provided in Figure 3.

### 2.5. Data Extracton and Synthesis

The initial data extraction is applied to all selected publications, and the following information was obtained:

- Titles and abstract of the included literature;
- Authors names;
- Publication year;
- Database;
- Region where the study was conducted (focused geographical area/country);
- Types of sensors used, and parameters analyzed (RQ1 and RQ2);
- Preferred MCUs, interfaces, and communication technologies (RQ3, RQ4, and RQ5);
- Analysis of power requirements and energy efficiency of the existing systems (RQ6);

Functionality details of the included studies (RQ7).

After data extraction, the synthesis procedure was applied to all selected studies to analyze and answer the research questions defined in Section 2.1. For RQ1 and RQ2, the different types of sensors used for monitoring IAQ conditions are listed, and their respective measured parameters are analyzed. For RQ3, an analysis about the preferred MCUs for designing a sensor network is reported, whereas for RQ4 and RQ5, the preferred interfaces and communication technologies are listed. For RQ6, we analyzed the power requirements of the different sensor networks included in this study. Functionality details of the included studies were analyzed for RQ7. Moreover, an in-depth comparison of sensor usability, reliability, and measuring performance was made while identifying the gaps in the knowledge.

### 2.6. Risk of Bias

The main limitation of this systematic review is that it is influenced by bias. The first biggest risk of bias arises in initiating the initial search query on the databases, as the results were limited to literature published after 2015. Moreover, the subjectivity of inclusion and exclusion criteria defined by the authors increases bias at the screening stage. Furthermore, the publications were obtained only from four databases (IEEE Explore, Web of Science, ScienceDirect, and PUBMED). Although they include some of the most reputable indexed databases from the academic field, the extensive range of publications from other databases such as Google Scholar, Scopus, and SpringerLink were not included.

## 3. Results

**Figure 4:**
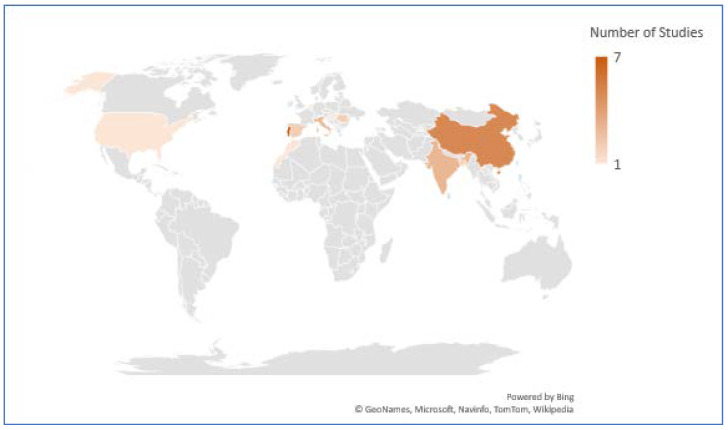
Distribution of studies from different locations of the world.

The rising threats of IAP with increased mortality and morbidity rates have motivated the researchers’ community to design numerous technology-inspired solutions to address the challenges. This systematic review includes 40 such studies based on IoT architecture from different parts of the world. In total, seven studies (17.5%) were conducted in Portugal, five studies (12.5%) were executed in China, and three studies (7.5%) were reported from India and Malaysia each. Furthermore, two studies (5%) were obtained from Indonesia, Romania, Spain, and Bangladesh each and one study each was reported from Singapore, Australia, Turkey, San Macros, Czech Republic, Korea, Hungary, Netherlands, USA, Morocco, and Qatar. Figure 4 shows the distribution of studies from different locations of the world.

The initial search query for this paper was conducted on four databases with a restriction on papers published before 2015. The distribution of the obtained 141 studies from different databases was discussed in Section 3.2. However, only 40 studies out of this list were included in this systematic review after applying pre-defined inclusion and exclusion criteria. Out of these, Web of Science contributed 21 studies, IEEE Explore provided 14 relevant studies, and 5 studies were included from the ScienceDirect database. However, none of the studies from the PUBMED database were found relevant as per the selection criteria of this systematic review. Table 2 shows the year-wise distribution of included studies from different databases.

The main aim of this systematic review is to provide insights into the existing IAQ monitoring systems that have been proposed by different researchers during or after 2015. There are numerous design architectures inspired by advanced technologies. However, this paper focuses on IoT-based designs only. The answers to RQ1 and RQ2 improve the understanding about potential IAQ parameters and types of sensors preferred for the measurements. The full-text screening included 40 studies providing details about 32 important IAQ and thermal comfort parameters that have been measured by existing researchers. Different researchers used numerous types of sensors to measure these IAQ parameters. However, these sensors can be mainly divided into four categories based on the scope of the measurement. Table 3 highlights details about the types of sensors used by different studies to measure the respective parameters.

### 3.1. Answer to RQ1 and RQ2

From the analysis of Table 3, it can be observed that 28 (70%) out of 40 studies preferred measuring two thermal comfort parameters (temperature and humidity). In total, 26 studies (65%) measured CO_2_ and 12 studies (30%) focused on measuring CO as critical IAQ parameters. Moreover, 11 studies (27.5%) included PM_10_ and PM_2.5_ sensors, and 8 studies (20%) measured volatile organic compounds (VOCs). This analysis reveals that temperature, humidity, CO_2_, CO, PM_10_, PM_2.5_, and VOCs are the most common monitored IAQ parameters. Additionally, 26 studies (65%) used 33 different types of dedicated sensors for IAQ monitoring, out of which 15 sensors were factory-calibrated. However, 14 studies (35%) used MQ series sensors (MQ135, MQ6, MQ4, MQ7, MQ9, MQ5, MQ2, MQ3) for measuring gaseous pollutants. Furthermore, 25% of the studies [61,63,68,70,78,79,81,82,86,90] used the MQ135 multi-gas sensor. The primary disadvantage of MQ series sensors is that they require field calibration. Moreover, the accuracy specifications are not defined in manufacturer datasheets.

All-in-one sensor boards were used by only two studies [83,85]. Since they can measure multiple parameters with in-built, pre-calibrated sensor probes, the higher cost of these sensors makes them unsuitable for real-time implementation. The commonly used sensors for thermal comfort measurement were DHT11 and DHT22 since they come in pre-calibrated form and offer a wide operational range (0–50 °C, 20–90% RH; −40 to +80 °C, 0 to 100% RH, respectively) for temperature and humidity measurement.

### 3.2. Answer to RQ3

Table 4 provides the distribution of MCUs used for the development of IAQ monitoring systems. Based on the results, the authors found that Arduino (37.5%) and Raspberry Pi (35%) were the most preferred slave and gateway MCUs. However, the most commonly used versions were Arduino Uno and Raspberry Pi. The ESP8266 module was used by 13 studies (32.5%), and it was commonly used as a gateway MCU, instead of slave MCU. All these three MCUs are available as open source platforms for real-time monitoring applications. Two studies also preferred using Waspmote as an MCU. However, the cost is the main concern for its implementation. One study [77] did not provide clear details about the used MCU for the slave or gateway operations.

### 3.3. Answer to RQ4

The preferred data consulting methods are presented in Table 5. It shows that 24 studies (60%) focused on the development of a mobile app for displaying the real-time status of the measured IAQ parameters. Moreover, 22 studies (55%) used the web portal/server to display IAQ characteristics. Two studies [67], [55] also preferred using an LCD display along with mobile apps to display the measured parameters. Furthermore, the authors of [73,88] did not provide clear details about preferred data consulting methods. Mobile apps provide a reliable solution for real-time measurements since they allow users to stay up to date regarding IAQ conditions anywhere and anytime. In addition, most of the web-based solutions require login before checking parameter updates. The LCD display along with mobile applications are a reliable alternative since they provide on-site updates and off-site tracking solutions as well.

Table 6 provides details about the preferred data storage methods of the analyzed studies. The results show that 26 studies (65%) preferred storing IAQ data on cloud servers since they provide easy access to IAQ updates from anywhere and anytime. Most of these researchers used Structured Query Language server databases for data storage. In total, eight studies (20%) used IoT data storage services for IAQ monitoring systems. In this case, ThingSpeak was the most preferred platform. Moreover, three studies each used local servers and SD cards or mobile internal storage for IAQ data. One study [85] did not provide any clear insights about the used data storage platform.

**Figure 5:**
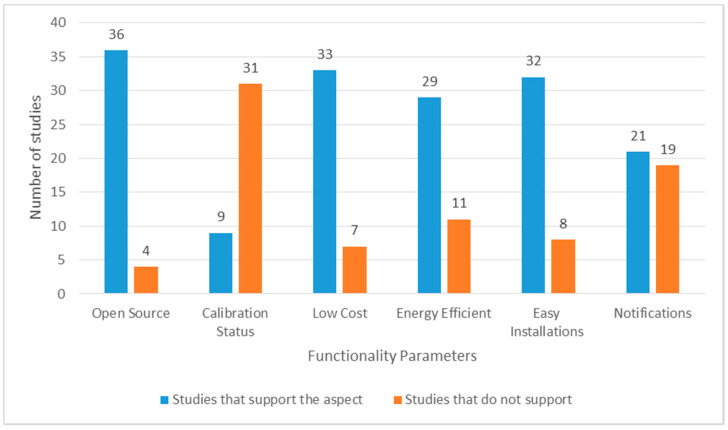
Functionality details of included studies.

Real-time alerts/notifications are a crucial feature of real-time monitoring systems. Table 7 provides insights into the preferred notification methods. The results show that 17 studies (42.5%) include mobile notifications to update users regarding significant variations in IAQ parameters. Moreover, six studies used SMS, and four studies included email-based alerts. Real-time alerts play a crucial role in preventing serious consequences associated with harmful IAQ levels. These notifications can provide active coaching for building occupants to take relevant interventions on time to improve ventilation and avoid negative IAQ exposure. However, only 21 studies (52.5%) (see Figure 5) include alerts features.

### 3.4. Answer to RQ5

From the analysis given in Table 8, it can be observed that Wi-Fi is the most preferred communication technology for IAQ monitoring systems. However, Bluetooth and ZigBee are the second and third most preferred technologies, respectively. In total, 28 studies (70%) used Wi-Fi for IAQ monitoring systems. However, 11 and 6 studies preferred Bluetooth and ZigBee, respectively. The main limitation of Wi-Fi for real-time monitoring application is the power consumption. Bluetooth and ZigBee are a reliable solution concerning low power consumption requirements. Furthermore, 13 monitoring systems (32.5%) that used Wi-Fi were based on ESP8266 MCU. However, nine monitoring systems (22.5%) were controlled by different versions of Raspberry Pi. The most preferred protocol for Wi-Fi communication is IEEE802.11 b/*n*/g, whereas IEEE 802.15.4 is used for ZigBee communication. Several researchers also preferred using the MQTT protocol due to its low power requirements and easy implementation as compared with IEEE 802.11.

### 3.5. Answer to RQ6

Another crucial parameter for performance analysis of IAQ monitoring systems is the energy consumption requirements. Table 9 shows that 17 studies (42.5%) preferred using the power supply, whereas 11 studies (27.5%) preferred using external batteries. Other than this, six and four studies used rechargeable battery and power banks, respectively. Solar cells have also been used for powering real-time monitoring systems as an evolutionary solution for green energy buildings. However, it was implemented by two studies only [62,84].

### 3.6. Answer to RQ7

The functionality details of the included studies are mentioned in Figure 5. Studies for which respective details were available and supported the mentioned performance parameter are indicated in blue. However, studies for which either concerned parameters are not true or the details are not defined in the manuscript are highlighted in orange. The results show that 36 studies (90%) worked on open source technologies and 33 studies (82.5%) preferred developing low-cost solutions for real-time implementation. Moreover, 32 (80%) and 29 studies (72.5%) were easier to install and included energy efficiency properties, respectively. However, only nine studies (22.5%) mentioned details about field calibration procedures before implementation.

These functionality details can be useful for future researchers to develop more relevant and impactful IAQ monitoring systems that can cover all essential design aspects.

## 4. Discussion

The above analysis conducted on 40 different studies shows that researchers have worked on an extensive range of IAQ parameters. However, the most preferred ones were temperature, humidity, CO_2_, CO, PM_10_, PM_2.5_, and VOCs. Furthermore, the studies are mainly focused on four types of sensors: thermal comfort sensors, multi-gas sensors, single gas sensors, and dust sensors. Nevertheless, only two studies used all-in-one sensor boards for IAQ measurement. Thermal comfort parameters are taken into consideration since they are closely associated with the comfort level of building occupants [91,92,93]. Several thermal comfort sensors are available in a pre-calibrated form, which makes them easy to use for real-time monitoring applications. However, before making the final selection, researchers need to consider the operating range of these sensors. CO_2_ and CO are two important gases that require the most attention from IAQ measurement applications since they are commonly found in rural as well as urban buildings [7,12,14]. The common sources of CO_2_ and CO emissions include unvented gas appliances, stoves, dryers, leaking furnaces/chimneys, wood stoves, fireplaces, and automobile exhausts from attached garages [94,95]. PM found indoors is originated from combustion, cooking, and candles and may include migrated particles from the outdoor environment [96,97]. The selection of focus IAQ parameters usually depends upon the target monitoring environment. For instance, the most common parameters for hospital measurement include temperature, humidity, CO, CO_2_, and VOCs [98]. Measurement in school buildings requires a focus on CO_2_, CO, O_3_, HCHO, VOCs, fungi, and bacteria [99,100]. For office buildings, primary parameters are temperature, humidity, CO_2_, CO, PM, VOCs, and microbial [101]. However, for residential buildings, more focus must be given to temperature, humidity, CO_2_, radon, and PM levels [102,103,104].

The selection of sensors for measuring IAQ parameters must be made based on several crucial factors such as cost, calibration requirements, and essential hardware needed for field implementation. Numerous sensors come in factory calibrated form, and they can be installed directly. However, several others need field calibration procedures. It is also crucial to check the nominal range of the sensors to ensure reliable results for specific monitoring conditions. The accuracy and response time specification of sensors are a crucial matter of concern for upcoming researcher activities. The correct selection of sensor units can provide reliable data for enhanced decision-making for occupant health and well-being.

On the one hand, the reviewed studies used different processing units. The most preferred options are Arduino, Raspberry Pi, and the ESP8266 module. On the other hand, Wi-Fi, Bluetooth, and ZigBee are commonly used communication technologies. Most of the studies used Arduino due to easy availability of support and documentation for this open source platform. The processing capabilities of Arduino Uno are restricted to 16MHz, and it does not provide in-built communication support. However, Raspberry Pi modules include an integrated communication module, and higher memory capacity and clock speed. Raspberry Pi 3 includes a 1.2GHz quad-core Arm Cortext-A53 processor. However, upcoming researchers can also use the Raspberry Pi 4 model with a 1.5GhZ 64-bit quad-core ARM Cortex-A72 processor. Furthermore, ESP8266 is a widely preferred Wi-Fi module for IoT applications due to its low-cost availability. There are three widely used configurations of ESP8266 boards. ESP-01 is the basic version which comes with 1MG flash memory, and it offers limited peripheral connectivity. Wemos D1 Mini and NodeMCU both offer 11GPIOs and 4MB flash memory. However, NodeMCU is the most used for IoT applications.

For real-time monitoring applications, power consumption is the main concern, and Wi-Fi-based operations are not always a reliable option. Consequently, several researchers are using Bluetooth and ZigBee platforms to provide power-efficient designs. However, the main disadvantage of these technologies is the limited communication range. In general, Bluetooth-based communication offers average coverage of 10 m, whereas ZigBee can be extended between 10 and 100 m [105]. However, ZigBee is widely preferred for its minimum energy consumption, and affordable cost of network setup [106]. In the future, it will be possible to integrate Wi-Fi modules with solar cells for green monitoring capabilities [62]. Solar cells can ensure a reliable solution for smart city and smart village projects while offering environment-friendly applications [107]. The developing countries are also working in the installation of 4G and 5G technologies which can play a critical role in the digital transformation of buildings [108]. The 5G technology can bring significant revolution in real-time monitoring applications with extended support to sensorial systems [15]. The advanced IAQ monitoring systems will be able to gather, analyze, transmit, and share data over big data platforms [15]. Such systems can involve enhanced decision-making possibilities for improved occupant health in buildings [109].

Healthy buildings need energy-efficient designs that can also reduce the cost of living [32]. It is crucial to work on the development of IAQ monitoring systems with low power consumption requirements [110]. The upcoming researchers should integrate monitoring systems with ventilation control mechanisms [43]. It is possible to influence the air exchange rate within buildings automatically depending upon the IAQ levels [111]. HVAC systems are an integral part of smart buildings [112]. However, they also cause a rise in PM levels [113]. Upcoming researchers should design highly automated systems for controlling the operation of HVAC systems in accordance with IAQ monitoring systems. Such sustainable approaches can promote a healthy lifestyle in urban areas. On the other side, it is relevant to design cost-efficient systems for rural areas. The low- and middle-income countries demand easy to install, energy-efficient, and affordable monitoring systems for promoting overall health and well-being [114]. Almost 2.5 billion people, one third of the population of the world, rely on biomass fuel for cooking and heating needs [115]. Therefore, it is crucial to design sustainable IAQ monitoring systems to prevent serious health concerns.

Mobile technologies have become an integral part of human life [116]. Therefore, new age monitoring systems must include smart applications to provide instant updates about IAQ levels [117]. The upcoming researchers need to focus on interactive app designs with additional features for data notification, visualization, and analytics. Forecasting of IAQ levels is another relevant concern for detecting unhealthy conditions in living environments [118]. It is crucial to design systems that can indicate potential threats in advance so that building occupants can take relevant control and preventive measures. These efforts can lead to sustainable smart buildings which promote public health and well-being to a considerable level [119]. Since people spend most of their time indoors, the development of energy-efficient, affordable, and sustainable IAQ monitoring systems can ensure inhabitants’ health and well-being.

Nevertheless, more focus is required on the development of easy to install and energy-efficient systems that can serve low-income populations. The upcoming researchers also need to study the development of a forecasting system with real-time alerts for IAQ conditions. These advanced systems can promote the efficient management of IAQ levels in smart buildings. Further initiatives must be taken to address the challenges associated with automated ventilation arrangements. It is crucial to work on the development of integrated systems that can control HVAC systems and natural ventilation sources in buildings. The novel technologies can support the enhanced monitoring and assessment of IAQ levels.

The findings of this study are relevant to support upcoming researchers, industry experts, public health policymakers, and governments. Furthermore, it is necessary to focus on the development of reliable IAQ monitoring features considering the current pandemic scenario. Recent studies reveal a close association of underlying respiratory health problems with COVID-19. Consequently, real-time monitoring of living environments promotes public health and well-being. These efforts can further prevent the morbidity and mortality rate due to associated underlying illnesses.

## 5. Conclusions

This systematic review presents the current state of the art of IAQ monitoring systems. This study includes 40 relevant studies of the last five years (2015–2020) obtained from four different databases. The majority of these studies (17.5%) were conducted in Portugal. However, other contributions were found located in China, India, and Malaysia. The results show that 70% of studies include temperature and humidity sensing as the main thermal comfort parameters. However, 65% of studies consider CO2 as a crucial IAQ parameter. Moreover, the preferred processing units for these IoT-based IAQ monitoring applications were Arduino (37.5%) and Raspberry Pi (35%), respectively. Wi-Fi communication is the widely preferred solution for internet connection followed by Bluetooth and ZigBee. In total, 90% of the researchers preferred using open source technologies for monitoring system implementation. However, the main limitation is the use of calibrated sensors. As the accuracy of the monitoring system is a crucial parameter for maintaining favorable living conditions, future researchers need to focus on adequate calibration arrangements. This analysis also opens opportunities for industry experts in designing calibrated sensors for real-time monitoring applications. Nevertheless, the current study also has limitations. The pre-defined inclusion and exclusion criteria limited the scope of the paper to IoT-based systems. Consequently, this systematic review does not provide details about WSN-based monitoring systems. Furthermore, the studies were obtained from four databases, and the restriction was applied for publications during and after 2015. In the future, the same study can be applied to study the progress in the field of outdoor air pollution.

## Figures and Tables

**Table 1:** Inclusion and exclusion criteria for systematic review.

Inclusion Criteria (IC)		Exclusion Criteria (EC)	
**IC1**	Papers that are based on IoT architecture.	EC1	Papers that are duplicates.
**IC2**	Papers that include “indoor” air pollution data	EC2	Papers that are based on outdoor air quality data.
**IC3**	Papers that provide insights into relevant IAQ parameters.	EC3	Papers that do not provide clear details about type of sensors used.
**IC4**	Papers that provide clear insights into system design methodology	EC4	Papers that are secondary studies.
**IC5**	Papers that are published after 2015.	EC5	Papers that are written in languages other than English.

**Table 2:** Year distribution of included studies from different databases.

Database	2015	2016	2017	2018	2019	2020
**Web of Science**	[53]	[54], [55]	[56], [57]	[52], [58], [59], [60], [61], [62], [63]	[64], [65], [66], [3], [67], [68], [69]	[70], [71]
**IEEE Explore**	-	-	[72], [73], [74], [75], [76]	[77], [78], [79]	[80], [81], [82], [83], [84]	[85]
**ScienceDirect**	-	-	-	[86], [87], [88], [89]	[90]	-

**Table 3:** IAQ sensors and relevant parameters discussed in existing studies.

Preferred Parameters	Thermal Sensors	Multi-Gas Sensors	Single Gas Sensors	Dust Sensors	All-in-One Sensor Board
Temp	[52], [72], [86], [54], [59], [87], [73], [60], [53], [80], [74], [88], [70], [77], [3], [90], [57], [62], [79], [69], [55], [63], [89]	[66], [84], [67]	-	-	[83], [85]
RH	[52], [72], [86], [54], [59], [87], [73], [60], [53], [80], [74], [88], [70], [77], [3], [90], [57], [62], [79], [69], [55], [63], [89]	[66], [84], [67]	-	-	[83], [85]
CO_2_	-	[81], [82], [62], [79]	[52], [54], [59], [87], [73], [56], [53], [80], [74], [88], [77], [3], [71], [57], [67], [78], [68], [69], [55], [89]	-	[83], [85]
CO	-	[64], [69],	[52], [54], [61], [77], [3], [82], [90], [78], [76]	-	[85]
PM_10_	-	-	-	[58], [72], [86], [87], [61], [70], [81], [3], [82], [57], [69]	-
PM_2.5_	-	-	-	[58], [72], [86], [87], [60], [80], [61], [70], [77], [3], [82]	-
VOCs	-	[66], [3], [60], [75], [67], [62], [87]	-	-	[83]
NO_2_	-	[64], [69]	[52], [61], [3]	-	-
O_3_	-	-	[52], [61], [77], [3]	-	-
LPG		[65], [86], [75], [76]			
AQI		[86], [84], [90], [63]			
Atm Pressure	[88], [89]	[84], [67]			
NH_3_		[64], [78]			[85]
C_3_H_8_		[64], [65]			
C_4_H_10_		[64], [65], [86]			
C_2_H_6_O					[85]
H_2_		[64], [76]			
SO_2_			[52], [61]		
C_6_H_6_		[76]	[3]		
CH_4_		[64], [76]	[75], [86]		
C_2_H_5_OH		[64], [75], [76]			
O_2_			[78]		[85]
Combustible gases		[74], [76]			
Air pressure		[66]			[83]
CL_2_			[52],		
PM_1.0_				[58]	
Noxious gases		[70]			
HCHO			[80], [77], [3]		
C_6_H_14_		[76]			
H_2_S					[85]
C_7_H_8_					[85]
Air velocity	[87]				

Temp = temperature, RH = relative humidity, CO_2_ = carbon dioxide, CO = carbon monoxide, PM_10_ = particulate matter (<10μm), PM_2.5_ = particulate matter (<2.5μm), VOCs = volatile organic compounds, NO_2_ = nitrogen dioxide, O_3_ = ozone, LPG = liquid petroleum gas, AQI = air quality index, Atm Pressure = atmospheric pressure, NH_3_ = ammonia, C_3_H_8_ = propane, C_4_H_10_ = butane, C_2_H_6_O = ethanol, H_2_ = hydrogen, SO_2_ = sulfur dioxide, C_6_H_6_ = benzene, CH_4_ = methane, C_2_H_5_OH = ethanol, O_2_ = oxygen, CL_2_ = chlorine, PM_1.0_ = particulate matter (<0.1μm), HCHO = formaldehyde, C_6_H_14_ = 2-Methylpentane, H_2_S = hydrogen sulfide carbonyl sulfide, C_7_H_8_ = methyl benzene.

**Table 4:** Microcontrollers used to connect sensors.

Microcontrollers	Ref	Number of Studies
ESP8266	[64], [59], [61], [88], [90], [57], [78], [82], [68], [69], [58], [54], [66]	13
ESP32	[71]	1
Arduino Nano	[86]	1
Arduino Uno	[87], [53], [61], [70], [81], [57], [67], [79]	8
Arduino Pro Mini	[72], [60]	2
Arduino Mega	[54], [59], [56], [78]	4
Raspberry Pi	[86], [73], [70], [81], [3], [62], [89]	7
Raspberry Pi2	[52], [72], [63]	3
Raspberry Pi3	[59], [74], [84]	3
Raspberry Pi3B+	[67]	1
Sunspot Module	[65]	1
STM32F103C8T6 (ARM)	[80]	1
Texas Instruments CC3200	[75]	1
ARM Cortex-M0	[62]	1
Waspmote	[52], [85]	2
EFR32 Mighty Gecko Wireless SoC	[83]	1
Intel Edison Board	[76]	1
MSP430F5529	[55]	1

**Table 5:** Preferred data consulting methods

Preferred Interfaces	Ref	Number of Studies
Web Portal/Server	[64], [52], [58], [72], [86], [54], [66], [59], [87], [60], [80], [61], [81], [3], [71], [83], [84], [62], [78], [76], [63], [89]	22
Mobile App/Smartphone	[64], [54], [66], [60], [53], [80], [61], [74], [70], [77], [81], [3], [71], [82], [84], [90], [57], [75], [67], [76], [68], [79], [69], [55]	24
Facebook API	[65]	1
Desktop App	[56],	1
LCD Display	[67], [55]	2

**Table 6:** Preferred data storage.

Preferred Interfaces	Ref	Number of Studies
Cloud server	[58], [72], [65], [54], [66], [59], [60], [53], [80], [61], [74], [88], [70], [77], [81], [3], [71], [82], [83], [84], [67], [62], [78], [68], [69], [89]	26
Local server	[56], [76], [79]	3
SD card/mobile internal storage	[53], [75], [55]	3
IoT datastore service	[64], [52], [86], [87], [73], [90], [57], [63]	8

**Table 7:** Preferred notification methods.

Preferred Options	Ref	Number of Studies
Mobile notifications	[64], [65], [54], [66], [60], [53], [80], [61], [74], [77], [3], [71], [82], [68], [79], [69], [85]	17
SMS	[72], [65], [70], [3], [55], [89]	6
Email	[58], [61], [55], [63]	4

**Table 8:** Preferred communication technologies used for IAQ monitoring systems.

Communication Technologies	Ref	Number of Studies
Wi-Fi	[64], [58], [72], [86], [66], [59], [73], [60], [53], [80], [61], [74], [88], [70], [77], [81], [82], [83], [90], [57], [75], [67], [78], [76], [68], [69], [85], [63]	28
Bluetooth	[65], [54], [73], [61], [53], [74], [71], [83], [84], [62], [79]	11
ZigBee	[52], [65], [54], [87], [56], [61]	6
LoRa	[60], [80]	2
GSM	[55]	1
GPS	[86]	1
GPRS	[80]	1
Ethernet	[85]	1

**Table 9:** Preferred sources of power to run IAQ monitoring systems.

Power Requirements	Ref	Number of Studies
Power supply	[52], [58], [72], [66], [56], [60], [53], [80], [74], [70], [77], [81], [82], [90], [67], [69], [63]	17
Power bank	[64], [88], [68], [69]	4
External battery	[65], [86], [59], [87], [73], [60], [71], [75], [55], [85], [89]	11
Rechargeable battery	[52], [54], [61], [83], [57], [78]	6
Solar cell	[84], [62]	2
